# Benzodiazepines and risk of dementia – Is there a reason for alarm?

**DOI:** 10.1192/j.eurpsy.2024.1319

**Published:** 2024-08-27

**Authors:** R. P. L. Andrade, N. Castro, R. Vaz, J. Martins, J. Abreu, E. Almeida, I. Santos, F. Cunha, H. Afonso

**Affiliations:** Centro Hospitalar Tondela-Viseu , Viseu, Portugal

## Abstract

**Introduction:**

The population ageing is a reality associated with an increase in prevalence of Dementia. The use of benzodiazepines is often postulated as a risk factor in these syndromes.

Contrary to recommendations for its short-time use, long-term and chronic use are common, with an estimated 8,7% of elderly people in the US taking benzodiazepines.

**Objectives:**

To clarify the most recent evidence on the use of benzodiazepines and the risk of developing dementia.

**Methods:**

Non-systematic review of literature, using PubMed as database and filtering the results for meta-analysis.

**Results:**

Four articles were included in this review.

Zhong G et al. concluded that risk of dementia increased in consumers of benzodiazepines and it was associated with higher doses.

In turn, AlDawasari A et al., when trying to clarify the use of different sedative-hypnotic drugs, found and increased risk with the consumption of benzodiazepines. After exclusion of articles with confounders and adjustment for protopathic bias, the risk was not maintained.

Lucchetta RC et al. concluded that the risk exists but without inferring differences between doses or duration of action.

Finally, Penninkilampi R e Eslick GD investigated this association, after controlling for the protopathic bias, concluding, contrary to AlDawasari et al., that the association benzodiazepines consumption and dementia do not result from this bias.

**Conclusions:**

We cannot draw robust and concrete conclusions between benzodiazepines consumption and the pathogenesis of dementia because not only is the literature limited, but results are also heterogeneous.

However, these prescriptions must be carried out cautiously, especially in the elderly, due to the known adverse effects associated with them.

**Disclosure of Interest:**

None Declared

